# Palmitic acid activates c-Myc via dual palmitoylation-dependent pathways to promote colon cancer

**DOI:** 10.1038/s41421-026-00869-6

**Published:** 2026-02-17

**Authors:** Wenxin Du, Jianing Zhang, Yuexin Wang, Minjun Li, Ji Cao, Bo Yang, Qiaojun He, Xuejing Shao, Meidan Ying

**Affiliations:** 1 Institute of Pharmacology and Toxicology, Zhejiang Province Key Laboratory of Anti-Cancer Drug Research, College of Pharmaceutical Sciences, Zhejiang University, Hangzhou, Zhejiang China; 2 Nanhu Brain-computer Interface Institute, Hangzhou, Zhejiang China; 3 School of Medicine, Hangzhou City University, Hangzhou, Zhejiang China; 4 Cancer Center, Zhejiang University, Hangzhou, Zhejiang China

**Keywords:** Colorectal cancer, Post-translational modifications, Transcriptional regulatory elements, Cancer metabolism, Targeted therapies

## Abstract

c-Myc is broadly hyperactivated in colon cancer, yet the mechanisms sustaining its transcriptional activation remain elusive. Here we identify palmitic acid (PA) as a metabolite cue that activates c-Myc via dual palmitoylation-dependent pathways operating across tumor initiation and progression. In colitis models, PA-rich diets exacerbate inflammation and enrich MYC target programs without increasing *Myc* mRNA. Mechanistically, the palmitoyltransferase ZDHHC9, upregulated by IL-1β, directly palmitoylates c-Myc at C171, enhancing c-Myc/MAX dimerization and transcriptional activity; genetic or pharmacologic inhibition diminishes c-Myc palmitoylation and target gene expression. During tumor progression, c-Myc transactivates FATP2, increasing PA uptake and reinforcing c-Myc palmitoylation, thereby establishing a feedforward loop and metabolic addiction to PA. Functionally, PA accelerates xenograft growth, whereas targeting ZDHHC9 and FATP2 inhibits c-Myc function to suppress tumor burden. These findings uncover metabolite-driven control of c-Myc through palmitoylation and highlight ZDHHC9/FATP2 as actionable vulnerabilities for colon cancer treatment.

## Introduction

Colon cancer represents as the third most common malignant tumor globally and the second leading cause of tumor-related deaths^1^. Deeply understanding the molecular processes involved in colon tumorigenesis and malignancies is important to find the novel therapeutic strategies for patients with colon cancer. As a powerful transcription factor, c-Myc, governs various essential cellular processes including cell proliferation, cell cycle, cell survival, cell stemness and others^2^. It is well acknowledged that c-Myc plays a critical role throughout the development of colon cancer. c-Myc is abnormally activated in colonic tissues with chronic inflammation^3,4^, which represents as the key driving event during the transition of inflammation-to-tumorigenesis^5,6^. Moreover, without regard to the development stages of colon cancer, almost all clinical samples of colon cancer exhibit varying degrees of abnormal transcriptional activation of c-Myc^7,8^, which manipulates the transcription of multiple downstream target genes, thereby promoting malignant phenotypes such as aggressive proliferation, tumor metastasis, cancer stemness, and chemotherapy resistance^9^. However, the mechanisms underlying the transcriptional hyper-activation of c-Myc remain largely unknown.

Metabolites function as signaling molecules to regulate various biological processes. Colon cancer is characterized by its location in the complex intestinal microenvironment, which inevitably leads to constant exposure to various metabolites. Metabolites, especially microbial metabolites have been widely known to regulate colon cancer. For example, deoxycholic acid generated from gut microbiota could promote intestinal barrier damage and tumorigenesis in the *APC*^min^ mouse model^10,11^. Meanwhile, dietary patterns and specific dietary components also found to play important roles in the tumorigenesis and progression of colon cancer. Yet the effects of metabolites on the transcriptional activation of c-Myc remain unidentified. It is well known that high-fat dietary intake and diet-derived various metabolites closely correlate with aggravated risk of colon cancer^12^. As the important component of high-fat diets, palmitic acid is not only an energy source for the body but also acts as a signaling molecule that widely participates in intracellular signal transduction networks^13,14^. We hypothesized that metabolites derived from high-fat diets such as palmitic acid might exert potential impacts on the regulation of c-Myc transcriptional functions.

Palmitoylation is a post-translational modification in which palmitoyl-CoA is covalently attached to the amino acid residues of proteins. This process is mainly catalyzed by 23 members of the zinc finger DHHC-type (ZDHHC) palmitoyltransferases family^15,16^. Protein palmitoylation can affect the function, localization, secretion, and stability of proteins by altering their lipid affinity. The involvement of abnormal palmitoylation in the pathological process of tumors and other diseases has been widely reported^17–19^. Palmitic acid mediates palmitoylation through its conversion into palmitoyl-CoA, and abnormal changes in its concentration may lead to dysregulated protein palmitoylation within cells^20^. Currently, no studies have reported whether c-Myc undergoes palmitoylation and whether palmitoylation as well as palmitic acid affects its transcriptional functions.

Dynamic adaptability of metabolism during the initiation and progression of cancers has been well acknowledged^21^, hence cancer cells display switched metabolic phenotypes to proactively adapt to changed metabolic demands^22,23^. Fatty acids such as palmitic acid are very important for energy storage and production of signaling molecules. c-Myc has been emerged as a key regulator that promotes both the synthesis as well as β-oxidation of fatty acids in order to fit the switched metabolic pattern of fatty acids. For example, c-Myc collaborates with SREBP1 to activate de novo synthesis of fatty acids, thereby facilitating energy production and malignant progression^24,25^. Until now, c-Myc was discovered to enhance the uptake of glucose, glutamine, essential amino acids^26^, but whether it could promote the uptake of fatty acids remains undetermined. Additionally, in neuroblastoma, N-Myc was reported to lead to glycerolipid accumulation by promoting fatty acid uptake and biosynthesis^27^. Thus, we wonder whether c-Myc could proactively promote the uptake of fatty acids such as palmitic acid in colon cancer.

Here, we have, for the first time, proposed a novel model in which metabolite can regulate the transcriptional activity of c-Myc. We find that palmitic acid activates c-Myc via dual palmitoylation-dependent pathways to promote colon cancer under distant metabolic patterns during tumorigenesis and cancer progression. Moreover, based on the metabolic vulnerability to palmitic acid in colon cancer, we propose a novel therapeutic strategy targeting palmitoyltransferase ZDHHC9 and fatty acid transporter FATP2. Collectively, our findings not only reveal a crucial biological role of palmitic acid on transcriptional activation of c-Myc, but also uncover a promising therapeutic option for patients with colon cancer.

## Results

### Palmitic acid promotes colonic inflammation and further facilitates c-Myc transcriptional activation

**Fig. 1:**
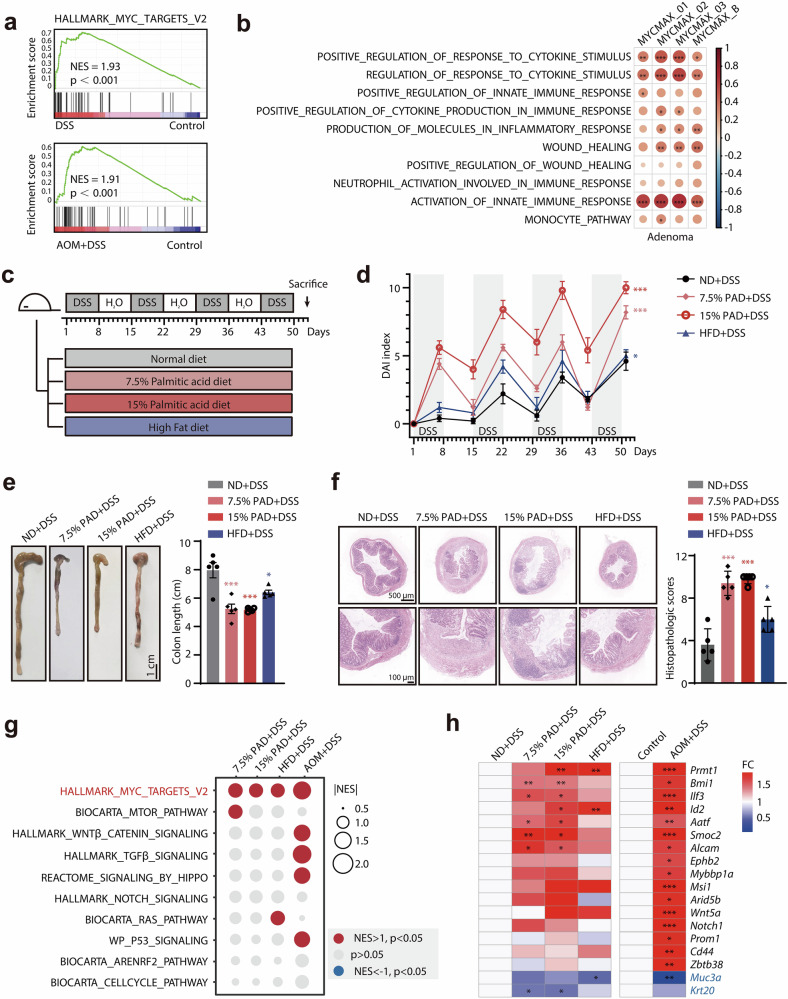
Palmitic acid promotes colonic inflammation and further facilitates c-Myc transcriptional activation. **a** Gene set enrichment analysis (GSEA) demonstrating the significant transcriptional activation of c-Myc in the DSS-induced colitis model (GSE208395) and murine model of inflammation-driven tumorigenesis treated with AOM and DSS (GSE249124). **b** The correlation of enrichment of signaling pathways involved in inflammation response and tissue repair with c-Myc transcriptional activity in adenoma samples from GSE117606 dataset. **c** Schematic diagram of experimental model of mice exposed to DSS-induced colitis respectively fed with ND, 7.5% PAD, 15% PAD and HFD. **d** DAI in each group. **e** Representative images of the colon tissue in each group. Colon length of each mouse was measured. **f** Representative H&E staining images of colonic sections in each group. Quantitative histopathologic analysis was performed. **g** GSEA of 10 canonical oncogenic pathways in colon tissues exposed to DSS treated with ND, 7.5% PAD, 15% PAD and HFD, as well as AOM + DSS induced tumor tissues versus paired normal colonic tissues (GSE249124). **h** Heatmap display of colonic stemness marker genes and epithelial differentiation marker genes in colon tissues exposed to DSS treated with ND, 7.5% PAD, 15% PAD and HFD, as well as AOM/DSS-induced tumor tissues vs paired normal colonic tissues (GSE249124). *p < 0.05; **p < 0.01; ***p < 0.001 vs indicated or control.

It is well acknowledged that inflammation promotes colon tumorigenesis, and we also confirmed the c-Myc activation during azoxymethane (AOM)/dextran sulfate sodium (DSS)-induced tumorigenesis, a well-established model for inflammation-associated colon cancer wherein AOM serves as an initiator carcinogen and DSS induces colonic inflammation (Fig. 1a; Supplementary Fig. S1). We found a positive correlation of c-Myc activity with inflammation response in colon adenomas (Fig. 1b), further indicating the potential role of c-Myc in inflammation-driven tumorigenesis. Given that high-fat diets promote colonic inflammation and -associated tumorigenesis^28–30^, we wonder whether high-fat diets as well as its important component palmitic acid could regulate c-Myc transcriptional functions. Mice were fed with normal diet (ND), a diet containing 7.5% or 15% palmitic acid diet (PAD) or classical high-fat diet (HFD), along with cyclic treatment of DSS (Fig. 1c). Compared to classical HFD (45%–60% Kcal), the 7.5% PAD mimics the palmitic acid contents in HFD (5.7%–8.5%, by mass), but lower lipid-derived caloric content (26% Kcal). In 15% PAD, palmitic acid almost serves as the sole lipid source to replace all fats in a classical HFD, while maintaining an equivalent fat-derived caloric fraction (45% Kcal) (Supplementary Fig. S2a). We first confirmed that PAD significantly increased palmitic acid levels in colonic tissues (Supplementary Fig. S2b). Notably, compared to the normal diet group, HFD as well as palmitic-acid-rich diet groups showed more obvious colonic inflammation under DSS exposure, as assessed by disease activity index (DAI) score (Fig. 1d). Consistently, similar to HFD group, mice in 7.5% PAD and 15% PAD groups presented significantly shortened colons (Fig. 1e) and serious inflammatory cell infiltration along with deteriorated epithelial defects and crypt atrophy (Fig. 1f). Additionally, mice fed with PAD and HFD showed severe bleeding as indicated by decreased red blood cell count, hemoglobin and hematocrit (Supplementary Fig. S2c). The high levels of circulating neutrophils and enlarged spleens in mice treated with PAD and HFD implied exacerbated colonic inflammation (Supplementary Fig. S2d, e). Moreover, palmitic acid alone could promote a modest increase in the DAI score and a slight worsening of hemorrhagic symptoms (Supplementary Fig. S3). These data suggested that the administration of palmitic acid could trigger colonic inflammation.

We further conducted RNA-sequencing of colonic tissues with chronic inflammation, and found that the MYC target signal was significantly enriched in the PAD as well as HFD groups, which is similar to that observed in the AOM + DSS tumorigenesis model^31^ (Fig. 1g). Correspondingly, we found that colonic cells in the PAD and HFD groups exhibited enhanced stemness and de-differentiation characteristics, as evidenced by the upregulation of genes such as *Bmi1* and *Ilf3*, and the downregulation of intestinal epithelial differentiation markers such as *Muc3a* and *Krt20* (Fig. 1h). These transcriptomic alterations are similar to inflammation-driven tumorigenesis model induced by AOM + DSS. Taken together, these results demonstrate that the important component of a high-fat diet, palmitic acid, could promote colonic inflammation, along with transcriptional activation of c-Myc.

### ZDHHC9 acts as an important factor regulating c-Myc transcription function during colon tumorigenesis

**Fig. 2:**
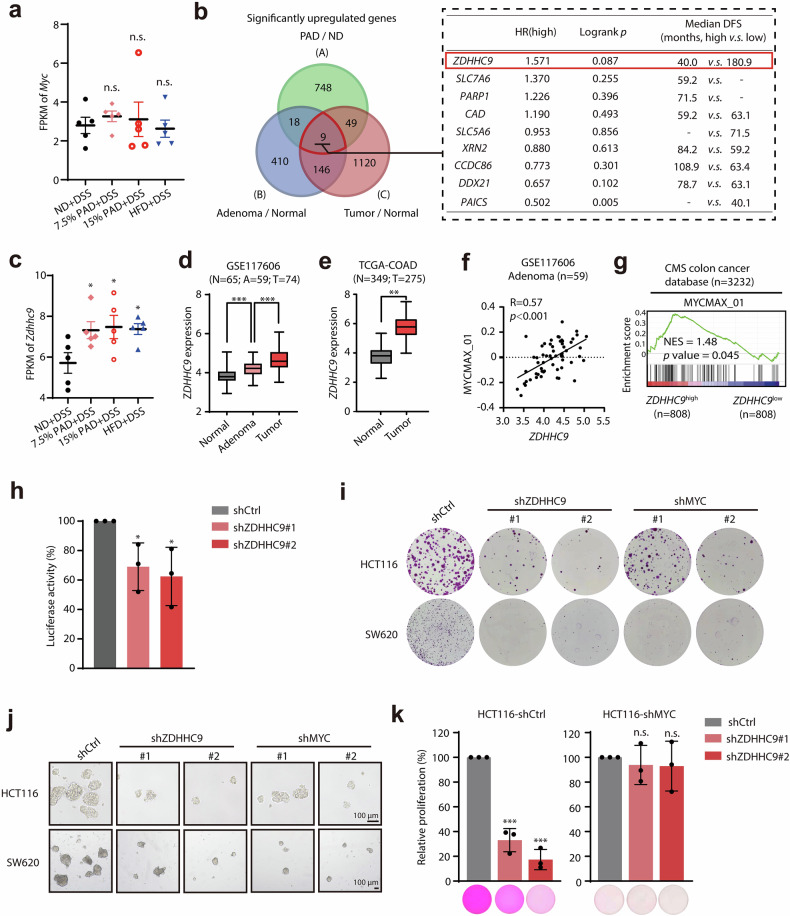
ZDHHC9 acts as an important factor regulating c-Myc transcription function during colon tumorigenesis. **a** The mRNA levels of *Myc* in colonic tissues from mice with DSS-induced colitis supplied by ND, 7.5% PAD, 15% PAD or HFD. **b** Left, overlapping analysis displayed 9 genes potentially manipulating c-Myc transcriptional activity in colon tumorigenesis. Cluster A: upregulated genes in colitis tissues exposed to 7.5% PAD and 15% PAD with abnormally enhanced c-Myc transcriptional activity compared to the ND group were represented by the green circle. Cluster B and C: upregulated genes which positively correlated with c-Myc transcriptional activity (quantitatively assessed by GSVA scores of c-Myc transcriptional target gene sets) in adenoma samples (GSE117606) and tumor samples (TCGA) were represented by the blue and red circles, respectively. Right, disease-free survival analysis of 9 candidate genes in colon cancer patients from TCGA cohort. Patients were divided into two groups with high expression or low expression of each candidate gene separately. HR, hazard ratio. **c** Analysis of *Zdhhc9* expression in colonic tissues from mice with DSS-induced colitis supplied by ND, 7.5% PAD, 15% PAD or HFD. **d** Analysis of *ZDHHC9* expression in adjacent normal tissues, adenoma tissues, and tumor tissues in GSE117606 dataset. **e** Analysis of *ZDHHC9* expression in normal samples and tumor samples from the GEPIA database. **f** Pearson correlation analysis of *ZDHHC9* expression with c-Myc transcriptional activity indicated by the GSVA score of MYCMAX_01 gene set in adenoma samples from GSE117606 dataset. **g** Gene set enrichment analysis demonstrating the significant positive correlation between *ZDHHC9* expression and c-Myc target genes in the CMS cohort^22^. **h** The inhibitory effect of ZDHHC9 knockdown on c-Myc transcriptional activity. **i** The colony formation assay showing the suppressed colony formation capacity caused by ZDHHC9 or MYC knockdown in colon cancer cell lines HCT116 and SW620. **j** The sphere formation assay showing the impaired sphere formation ability caused by ZDHHC9 or MYC knockdown in colon cancer cell lines HCT116 and SW620. **k** The cell proliferation assay showing the inhibitory effects of ZDHHC9 knockdown in colon cancer cells HCT116 infected with lentivirus-shCtrl or shMYC#2, as evaluated by SRB staining. n.s., *p* > 0.05; *p < 0.05; **p < 0.01; ***p < 0.001 vs indicated or control.

In order to further uncover the underlying mechanisms of c-Myc transcriptional activation during palmitic acid-induced chronic inflammation, we first checked whether the mRNA levels of *Myc* were upregulated. Surprisingly, compared to ND group, two PAD groups as well as HFD group that showed activation of c-Myc signal did not show significantly higher expression of *Myc* gene (Fig. 2a), indicating that there may be potential regulators manipulating transcriptional activity of c-Myc during this process. To identify potential regulators, we first determined the up-regulated genes in the PAD as well as HFD groups (Cluster A), and in the colon adenoma samples (Cluster B) as well as colon cancer specimens (Cluster C). Through overlapping analysis, 9 genes were identified (Fig. 2b, left). Among them, only palmitoyltransferase encoding gene *ZDHHC9* displayed the positive correlation with poor prognosis in colon cancer patients, while other 8 genes did not (Fig. 2b, right). Significantly elevated *ZDHHC9* expression was demonstrated in PAD- and HFD-treated inflamed tissues (Fig. 2c), and was also observed in an inducible murine model of inflammation-driven tumorigenesis (Supplementary Fig. S4a).

Next, we further investigated the relationship between ZDHHC9 and c-Myc transcriptional activation during colon tumorigenesis as well as progression. First, gradually increased mRNA level of *ZDHHC9* was also observed during the development of colon cancer (Fig. 2d, e). Moreover, the expression levels of *ZDHHC9* were positively correlated with the activation of c-Myc transcriptional functions in adenoma samples (Fig. 2f; Supplementary Fig. S4b). Meanwhile, in colon cancer, the transcriptional target genes of c-Myc were positively enriched in samples with higher *ZDHHC9* expression (Fig. 2g). The samples with similar level of c-Myc expression but higher transcriptional activity exhibit significantly upregulated expression of *ZDHHC9* (Supplementary Fig. S4c, d). These analyses indicated that *ZDHHC9* expression might be associated with c-Myc transcriptional activity. Then, we confirmed that ZDHHC9 knockdown significantly decreased the transcriptional activity of c-Myc (Fig. 2h). To further define the potential role of ZDHHC9 in colon cancer, we infected colon cancer cells with lentivirus packaging *shZDHHC9*. Notably, *shZDHHC9* potently impaired the proliferation, colony formation and sphere formation capacity of colon cancer cells, similar to the inhibitory effect of *shMYC* (Fig. 2i, j; Supplementary Fig. S5). In addition, proliferation assays revealed that ZDHHC9 knockdown significantly suppressed proliferation in HCT116-shCtrl cells but did not in HCT116-shMYC cells, suggesting that the function of ZDHHC9 might be dependent on c-Myc in colon cancer cells (Fig. 2k). Taken together, our results suggest that ZDHHC9 might act as an important factor regulating c-Myc transcription function during colon tumorigenesis.

### ZDHHC9 is upregulated by inflammation factor IL-1β

**Fig. 3:**
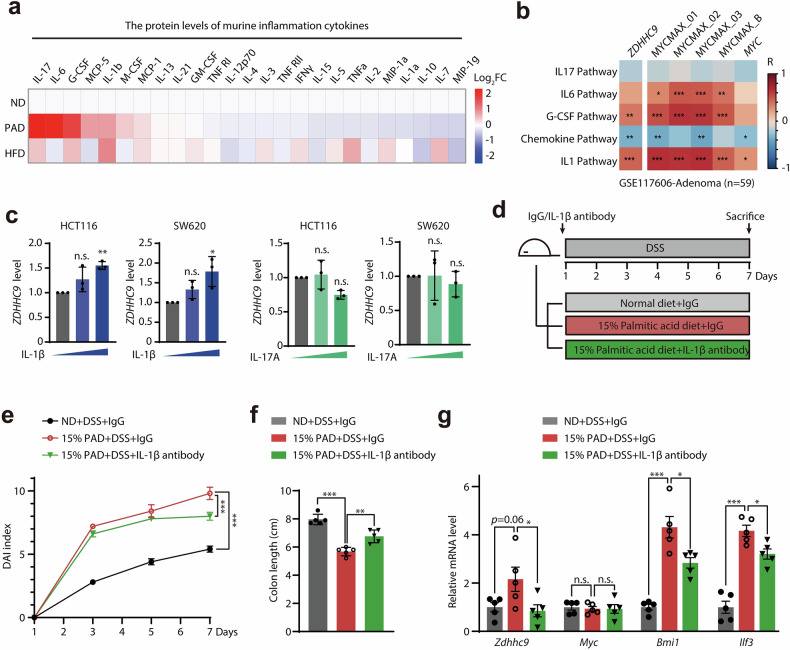
ZDHHC9 is upregulated by inflammation factor IL-1β. **a** Heatmap display of relative protein levels of inflammation factors in colon tissues exposed to DSS treated with ND, PAD and HFD. **b** The correlation of enrichment of signaling pathways mediated by inflammation factors with *ZDHHC9* expression as well as c-Myc transcriptional activity in adenoma samples from GSE117606 dataset. **c** The effects of inflammation factor IL-1β and IL-17A on *ZDHHC9* mRNA levels in colon cancer cells HCT116 and SW620. **d** Schematic diagram of experimental model of mice exposed to 2% DSS-induced colitis respectively fed with ND, 7.5% PAD and subjected to administration of IgG or IL-1β antibody (10 mg/kg, *i.p*.). **e** DAI in each group. **f** Colon length in each group. **g** *Zdhhc9*, *Myc*, *Bmi1* and *Ilf3* mRNA levels in each group. n.s., *p* > 0.05; *p < 0.05; **p < 0.01; ***p < 0.001 vs indicated or control.

Since ZDHHC9 was upregulated in colonic tissues with inflammation induced by PA, we were encouraged to explore the potential cause of ZDHHC9 upregulation during inflammation-associated tumorigenesis. We firstly detected the changes in the protein levels of inflammatory factors in colonic tissues treated with palmitic-rich diet through chip analysis, then found that the top 5 significantly upregulated inflammatory factors were IL-17A, IL-6, G-CSF, MCP-5, and IL-1β (Fig. 3a). The Pearson correlation analysis showed that the enrichment degree of IL-1β pathway was significantly positively correlated with *ZDHHC9* expression and c-Myc transcriptional activity, and the correlation was the strongest (Fig. 3b). Furthermore, we found that IL-1β treatment significantly upregulated the mRNA level of *ZDHHC9* in vitro, while IL-17A did not (Fig. 3c), indicating that the inflammatory factor IL-1β may stimulate *ZDHHC9* expression.

Furthermore, we sought to investigate whether IL-1β upregulates *ZDHHC9* expression in vivo. In mice with DSS-induced colitis, IL-1β neutralization partially reversed the aggravated colitis phenotypes promoted by PAD, as reflected by attenuated weight loss, lower disease activity index scores, and reduced colon shortening (Fig. 3d–f; Supplementary Fig. S6). More importantly, IL-1β blockade inhibited the transcriptional upregulation of *Zdhhc9* and MYC target genes related to stemness including *Bmi1* and *Ilf3*, without affecting *Myc* expression (Fig. 3g). Taken together, these data indicated that ZDHHC9 might be potentially upregulated by inflammatory factors including IL-1β, thus activating the transcription function of c-Myc and promoting colon tumorigenesis.

### ZDHHC9-catalyzed palmitoylation regulates c-Myc transcriptional activity

**Fig. 4:**
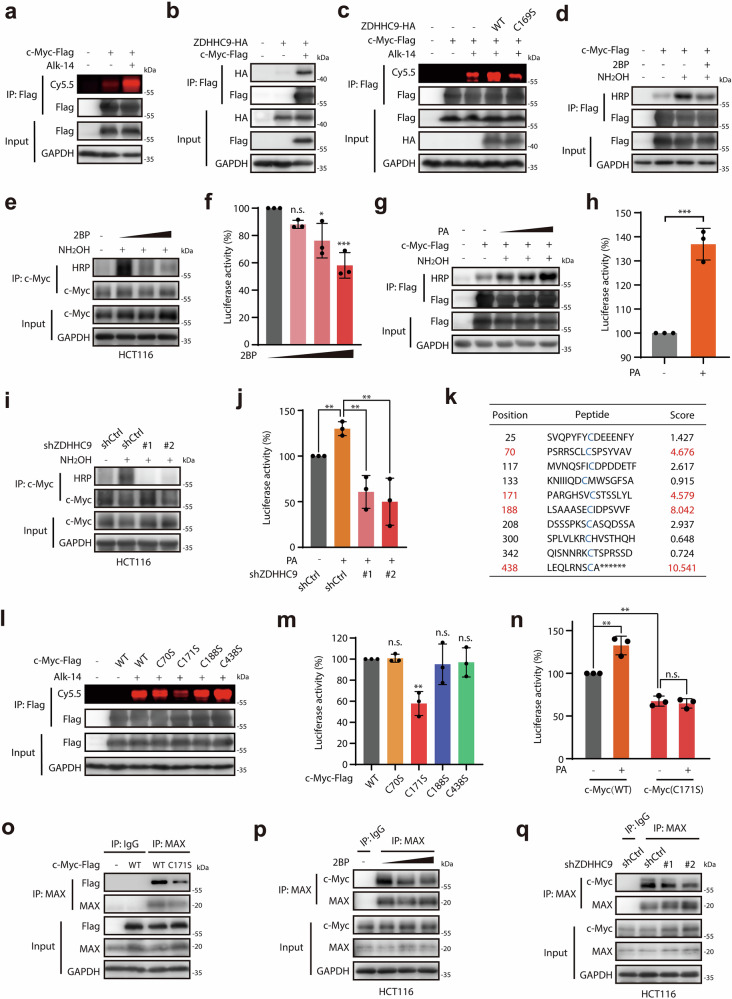
ZDHHC9-catalyzed palmitoylation regulates c-Myc transcriptional activity. **a** In-gel fluorescence corroborated significant palmitoylation of c-Myc protein. **b** The protein-protein interaction of ZDHHC9 with c-Myc. **c** In-gel fluorescence exhibited the effect of ZDHHC9-wild-type (WT) and ZDHHC9-C169S on the palmitoylation level of c-Myc protein. **d** ABE assay showed the inhibition of pan-palmitoyltransferase inhibitor 2BP (20 μM) on the palmitoylation level of exogenous c-Myc protein in COS-7 cells. **e** ABE assay showed the inhibition of pan-palmitoyltransferase inhibitor 2BP (20 μM or 40 μM) on the palmitoylation level of c-Myc protein in HCT116 cells. **f** The inhibitory effect of 2BP (20 μM, 40 μM or 80 μM) on c-Myc transcriptional activity. **g** ABE assay showed the promoting effect of palmitic acid (50 μM or 100 μM) on the palmitoylation level of exogenous c-Myc protein in COS-7 cells. **h** The promoting effect of palmitic acid (25 μM) on c-Myc transcriptional activity. **i** ABE assay showed the inhibition of ZDHHC9 knockdown on the palmitoylation level of c-Myc protein in HCT116 cells. **j** The inhibitory effect of ZDHHC9 knockdown on c-Myc transcriptional activity. **k** Cysteine sites C70, C171, C188, and C438 of c-Myc protein were predicted by CSS-palm 4.0 as potential palmitoylation sites. **l** In-gel fluorescence showed the palmitoylation level of c-Myc WT as well as C70S, C171S, C188S, and C438S mutant in COS7 cells. **m** The transcriptional activity of c-Myc WT as well as C70S, C171S, C188S, and C438S mutant. **n** The transcriptional activity of c-Myc WT and C171S mutant with the treatment of palmitic acid (25 μM). **o** Co-immunoprecipitation assay showed the interaction of c-Myc wild type and C171S mutant with MAX in HEK-293T cells. **p** Co-immunoprecipitation assay showed the interaction of c-Myc with MAX in HCT116 cells treated with 2BP (20 μM or 40 μM). **q** Co-immunoprecipitation assay showed the interaction of c-Myc with MAX in HCT116 cells infected with shCtrl, shZDHHC9#1 or shZDHHC9#2 lentivirus. n.s., *p* > 0.05; *p < 0.05; **p < 0.01; ***p < 0.001 vs indicated or control.

We further investigated the molecular mechanism of ZDHHC9 regulating c-Myc transcriptional activity. Acting as one of the palmitoyltransferase family members, ZDHHC9 could modulate functions of substrate proteins through catalyzing palmitoylation. Therefore, we hypothesized that ZDHHC9 might activate c-Myc transcriptional function by regulating its palmitoylation. As expected, the significant palmitoylation of c-Myc protein was confirmed by utilizing Alk-14 labeling combined with click chemistry (Fig. 4a). The physical interaction between ZDHHC9 and c-Myc protein was further validated (Fig. 4b). More importantly, we found that wild-type ZDHHC9, rather than its catalytically-deficient mutant (C169S), strongly increased palmitoylation of c-Myc protein (Fig. 4c), indicating that ZDHHC9 is a critical palmitoyltransferase of c-Myc.

Then we tried to determine whether palmitoylation regulates transcriptional function of c-Myc. Palmitoyltransferase inhibitor 2BP was introduced, and results showed that 2BP significantly reduced the palmitoylation level of c-Myc protein and decreased c-Myc transcriptional activity in a dose-dependent manner (Fig. 4d–f; Supplementary Fig. S7a, b). Furthermore, palmitic acid treatment increased c-Myc palmitoylation (Fig. 4g) and, in addition, enhanced its transcriptional activity (Fig. 4h). More importantly, ZDHHC9 knockdown significantly reduced the palmitoylation level of c-Myc protein and impaired c-Myc transcriptional activation induced by PA (Fig. 4i, j). Next, CSS palm 4.0 model was applied to predicting potential palmitoylation sites of c-Myc protein. As shown in Fig. 4k, four cysteine sites C70, C171, C188, and C438 presented relatively higher prediction scores, thus we respectively mutated four cysteine sites to serine (S) to investigate the palmitoylation level as well as transcriptional activity of these four mutants. Compared to wild-type protein, the palmitoylation level of c-Myc C171S mutant was significantly reduced, while the other three cysteine mutants displayed no significant difference (Fig. 4l), which indicated that C171 represented a potential palmitoylation site of the c-Myc protein. Consistently, overexpression of ZDHHC9 did not increase the palmitoylation level of the C171S mutant, in contrast to its effect on the wild-type protein (Supplementary Fig. S7c). Moreover, the mutation of C171 site, rather than other three cysteine sites, impaired the transcriptional activity of c-Myc (Fig. 4m, n; Supplementary Fig. S7d), and ZDHHC9 knockdown failed to decrease the transcriptional activity of the C171S mutant (Supplementary Fig. S7e), further suggesting that palmitoylation site C171 might be critical for c-Myc transcriptional function. These findings indicate that the transcriptional activation of c-Myc is highly dependent on the ZDHHC9-catalyzed palmitoylation.

Furthermore, we were encouraged to investigate how ZDHHC9-catalyzed palmitoylation regulate c-Myc transcriptional activity. A recent report identified a covalent ligand EN4, which targets cysteine 171 and might potentially disrupts MYC transcriptional activity by inhibiting MYC/MAX binding^32^. Thus, we postulated that palmitoylation at C171 site might regulate c-Myc transcriptional function by affecting its binding to MAX. Results showed that the C171S mutation impaired c-Myc/MAX binding (Fig. 4o). Consistently, both 2BP treatment and ZDHHC9 knockdown reduced endogenous c-Myc/MAX complex formation without altering c-Myc protein levels or subcellular localization (Fig. 4p, q; Supplementary Fig. S7f–h). These data indicated that the inhibition of c-Myc palmitoylation could potentially impact the c-Myc/MAX heterodimerization, followed by impaired c-Myc transcriptional activity.

In summary, we propose that at the inflammation-cancer transformation stage, palmitic acid might stimulate inflammation to upregulate ZDHHC9, which in turn enhances c-Myc transcriptional function through palmitoylation.

### c-Myc transactivates fatty acid transporter FATP2 causing addiction to palmitic acid in developed colon cancer

**Fig. 5:**
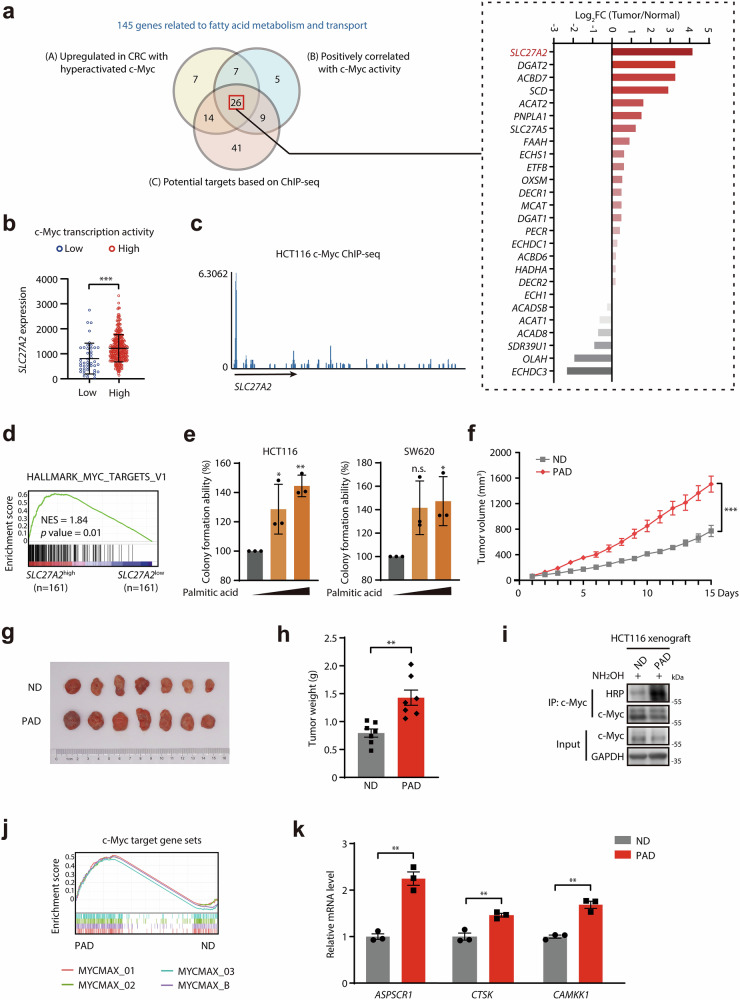
c-Myc transactivates fatty acid transporter FATP2 causing addiction to palmitic acid in developed colon cancer. **a** Left, venn diagram displaying 26 candidate genes related to fatty acid metabolism and transport which is highly associated with c-Myc transcriptional activity. The yellow circle indicated the significantly upregulated genes in the colon cancer group with relatively high transcription activity of c-Myc from TCGA cohort; The blue circle indicated the genes positively correlated with GSVA score of HALLMARK_MYC_TARGET_V1 gene set in colon cancer samples from TCGA cohort; The red circle indicated the putative transcriptional targets of c-Myc in colon cancer cell lines among 145 genes involved in fatty acid metabolism and transport. ChIP-seq data were obtained from GSE78064, GSE117240, and GSE51234 and then analyzed by Cistrome DB. Right, the fold changes of expressions of 20 candidate genes in colon cancer samples compared to normal samples from GEPIA database. **b** The expression of FATP2 in colon cancer groups with high and low c-Myc transcriptional activity from TCGA database. **c** The enrichment of c-Myc at *FATP2* promoter region in colon cancer cell HCT116. ChIP-seq data were obtained from GSE78064 and analyzed by Cistrome DB. **d** GSEA plot depicting the enrichment of c-Myc target genes in colon cancer samples with relatively higher *SLC27A2* expression. **e** The colony formation assay of colon cancer cells HCT116 and SW620 cultured in media containing fetal bovine serum with the treatment of palmitic acid (3.13 μM or 6.25 μM). **f**–**h** The effect of 7.5% PAD on tumor growth of HCT116 xenografts in nude mice. **f** The tumor growth of HCT116 subcutaneous xenografts in nude mice supplied by ND or 7.5% PAD. **g** The image of HCT116 xenografts in each group. **h** The tumor weights of HCT116 xenografts in each group. **i** ABE assay showed the promoting effect of PAD on the palmitoylation level of c-Myc protein in HCT116 xenografts. **j** GSEA plots depicting enrichment of c-Myc target gene sets in HCT116 xenografts obtained from nude mice supplied by 7.5% PAD. **k** The expression levels of c-Myc target genes in HCT116 xenografts obtained from nude mice supplied by ND or 7.5% PAD measured by RT-PCR. n.s., *p* > 0.05; *p < 0.05; **p < 0.01; ***p < 0.001 vs indicated or control.

c-Myc has emerged as a key regulator that promotes both the synthesis and oxidation of fatty acids. We wonder whether the hyperactivated c-Myc would, in turn, regulate fatty acid metabolism to promote colon cancer. First, we conducted an analysis of associations between c-Myc transcriptional activity (indicated by GSVA scores of c-Myc downstream gene sets) and expressions of 145 genes related to fatty acid metabolism as well as transport^33^ in colon cancer. As shown in Fig. 5a, we identified the upregulated genes in colon cancer with hyperactivated c-Myc (Cluster A), genes positively correlated with c-Myc activity (Cluster B) and potential targets of c-Myc based on c-Myc ChIP-seq in colon cancer cell lines (Cluster C). Among these 28 overlapped genes, FATP2 (encoded by *SLC27A2* gene), responsible for long-chain fatty acid uptake including palmitic acid, demonstrated the most upregulated expression in colon cancer specimens. In detail, FATP2 was upregulated in colon cancer samples with relatively high c-Myc transcriptional activation (Fig. 5b) and might be the direct transcriptional target of c-Myc according to ChIP-seq results (Fig. 5c), indicating that FATP2 might be potentially transactivated by c-Myc. Given that palmitic acid could promote palmitoylation and transcriptional activation of c-Myc, and GSEA revealed that colon cancer with higher level of FATP2 displayed activated c-Myc transcriptional signaling (Fig. 5d; Supplementary Fig. S8a), we suspect that transactivated FATP2 might enhance the palmitic acid uptake to further activate c-Myc.

Next, we investigated whether enriched palmitic acid could activate c-Myc and accelerate the development of colon cancer. The treatment with palmitic acid significantly enhanced the colony formation ability of colon cancer cells (Fig. 5e; Supplementary Fig. S8b). In vivo experiments further demonstrated that palmitic acid dramatically promoted the tumor growth in subcutaneous xenograft mouse models of HCT116 cells (Fig. 5f–h; Supplementary Fig. S8c), supporting that abnormally high levels of palmitic acid significantly promoted colon cancer progression. More importantly, we also observed that palmitic acid obviously increased the palmitoylation of c-Myc proteins in HCT116 xenografts (Fig. 5i), while transcriptional activation of c-Myc was consistently detected in tumors treated with palmitic acid (Fig. 5j, k). Collectively, these data indicated that c-Myc transactivates fatty acid transporter FATP2 followed by the accumulation of cellular palmitic acid, which might recruit a positive feedforward regulatory loop and provoke addiction to palmitic acid in colon cancer at the progression stage.

### Targeting ZDHHC9 and FATP2 exhibits strong therapeutic effects on colon cancer

**Fig. 6:**
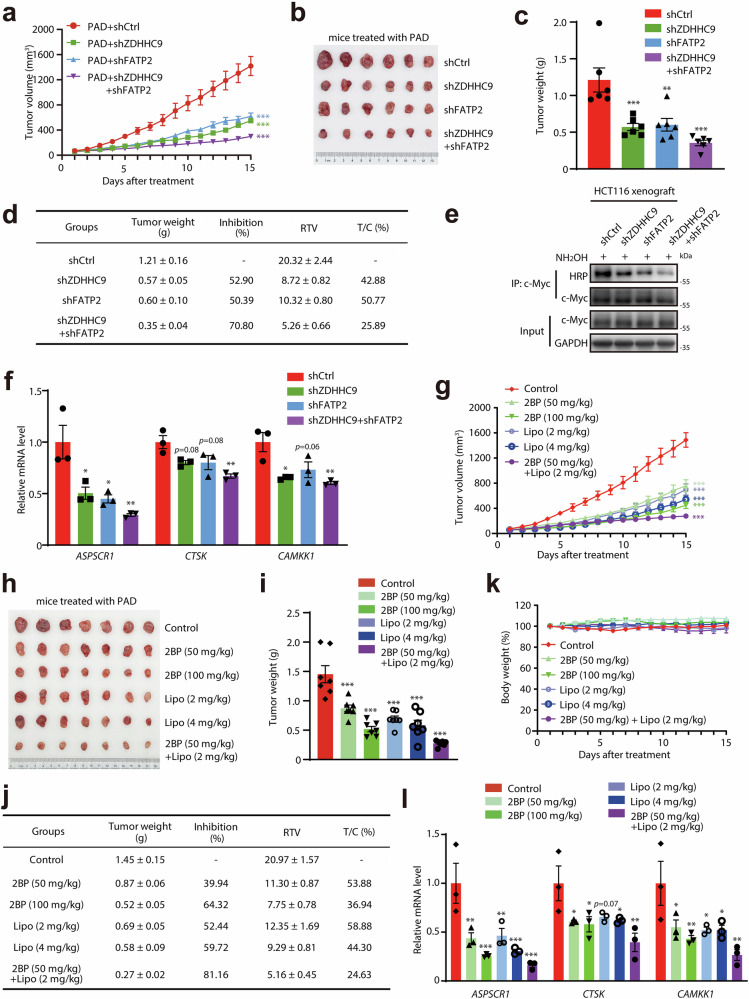
Targeting ZDHHC9 and FATP2 exhibits strong therapeutic effects on colon cancer. **a**–**d** The therapeutic effects of ZDHHC9 and FATP2 knockdown on the tumor growth of HCT116 xenografts in nude mice fed with 7.5% PAD. **a** The tumor growth of HCT116 subcutaneous xenografts in nude mice exposed to 7.5% PAD with intratumoral injection of shCtrl, shZDHHC9#2, shFATP2#1 and combination of shZDHHC9#2 with shFATP2#1 lentivirus. **b** The image of HCT116 xenografts in each group. **c** The tumor weights of HCT116 xenografts in each group. **d** The therapeutic effects of ZDHHC9 and FATP2 knockdown on HCT116 xenografts in nude mice fed with 7.5% PAD. **e** ABE assay showed the suppression effect of ZDHHC9 and FATP2 knockdown on the palmitoylation level of c-Myc protein in HCT116 xenografts. **f** The expression levels of c-Myc target genes in HCT116 xenografts in each group measured by RT-PCR. **g**–**k** The therapeutic effects of 2BP and Lipofermata on the tumor growth of HCT116 xenografts in nude mice fed with 7.5% PAD. **g** The tumor growth of HCT116 subcutaneous xenografts in nude mice exposed to 7.5% PAD with intraperitoneal administration of saline, 2BP (50 or 100 mg/kg), Lipofermata (2 or 4 mg/kg) and combination of 2BP (50 mg/kg) with Lipofermata (2 mg/kg). **h** The image of HCT116 xenografts in each group. **i** The tumor weights of HCT116 xenografts in each group. **j** The therapeutic effects of 2BP and Lipofermata on HCT116 xenografts in nude mice fed with 7.5% PAD. **k** The relative body weight of nude mice in each group. **l** The expression levels of c-Myc target genes in HCT116 xenografts in each group measured by RT-PCR. n.s., *p* > 0.05; *p < 0.05; **p < 0.01; ***p < 0.001 vs indicated or control.

Based on metabolic addiction to palmitic acid in colon cancer at the progression stage, we then asked whether targeting ZDHHC9 and FATP2 could achieve effective therapeutic treatment for colon cancer. We first evaluated in vivo therapeutic effects of ZDHHC9 and FATP2 knockdown through intratumoral injection of lentivirus in nude mice supplied with palmitic-acid-rich diet (Supplementary Fig. S9). As shown in Fig. 6a–d, ZDHHC9 and FATP2 depletion obviously attenuated HCT116 xenograft tumor growth promoted by PA. Notably, the combined knockdown of ZDHHC9 and FATP2 led to a markedly greater suppression (reaching 70.80%). Meanwhile, we confirmed that ZDHHC9 and FATP2 knockdown markedly reduced intratumoral c-Myc palmitoylation and the transcription of c-Myc target genes (Fig. 6e, f). These findings suggested that targeting ZDHHC9 and FATP2 could inhibit colon cancer progression through modulating c-Myc palmitoylation.

Encouraged by the in vivo inhibitory effects of ZDHHC9 and FATP2 knockdown, we next assessed anti-tumor activity of pan-ZDHHCs inhibitor 2BP and FATP2 inhibitor Lipofermata against colon cancer. Both 2BP and Lipofermata could potently inhibit the stemness potential of colon cancer cells in vitro, as reflected by significantly impaired sphere formation and colony formation abilities (Supplementary Fig. S10). Then we assessed therapeutic activities of pharmacological targeting ZDHHC9 and FATP2 on colon cancer in vivo. We observed that both 2BP and Lipofermata treatment arrested HCT116 xenograft tumor growth facilitated by PA (Fig. 6g–i). The combination treatment of 2BP and Lipofermata further remarkably alleviated tumor burden by 81.16% (Fig. 6j). It is noteworthy that 2BP or Lipofermata treatment did not show toxicity, as evaluated by body weight changes (Fig. 6k). Meanwhile, we also verified that the treatment of 2BP and Lipofermata reduced intratumoral PA contents (Supplementary Fig. S11) and consistently inhibited the transcriptional activation of c-Myc (Fig. 6l). Taken together, these findings demonstrated that targeting metabolic addiction to palmitic acid as a vulnerability through inhibition of ZDHHC9 and FATP2 displayed strong therapeutic effects against colon cancer.

## Discussion

Numerous studies have shown that almost all colon cancer samples exhibit abnormally enhanced c-Myc transcriptional activity. However, the level of c-Myc alone could not fully explain the widespread hyperactivity of c-Myc. Here, we propose a novel mechanism for manipulating c-Myc transcriptional activity. We found that the transcriptional function of c-Myc could be activated by ZDHHC9 through mediating palmitoylation of c-Myc at cysteine 171 and c-Myc/MAX binding. The regulatory effect of palmitoylation on several transcription factors has been studied. ZDHHC7 palmitoylates STAT3, promoting its membrane recruitment and phosphorylation, which further leads to activation^17^. Palmitoylation ensures the correct folding of TEAD protein and its interaction with YAP/TAZ^18,19^. Further studies are needed to investigate how palmitoylation regulates the assembly of the c-Myc/MAX heterodimeric complex. Although inhibition of palmitoylation reduced c-Myc/MAX dimerization, it remains unclear whether this is a direct effect of palmitoylation or a consequence of disrupted assembly of the c-Myc transcriptional complex. Further structure-based investigation is needed.

Currently, several studies suggest that ZDHHC9 plays a promotive role in tumorigenesis. For instance, ZDHHC9 promotes glioma progression by facilitating GLUT1 plasma membrane localization, thereby enhancing glycolytic flux^34^. Additionally, it catalyzes the palmitoylation of HRAS and NRAS, which is necessary for their affinity at the plasma membrane and RAS signaling^35^. Genetic ablation of ZDHHC9 significantly impedes malignant progression in various cancers such as glioma, leukemia, breast cancer and pancreatic cancer^36–39^. In our study, we proposed that ZDHHC9 might not function as a classical oncogene itself. Instead, it might collaborate with the established oncogene c-Myc to cooperatively promote tumorigenesis and development. However, it is undeniable that the functions of ZDHHC9 may vary under different physiological and pathological conditions depending on its substrates, and this warrants further investigation in the future.

Aroused focus is given to understanding the physiological and pathological mechanisms that connect various metabolites to the regulation of transcriptional factors. For instance, the dynamic changes in glucose concentration indirectly manipulate the phosphorylation, acetylation and O-GlcNAcylation of the transcription factor ChREBP, while these post-translational modifications influence the nuclear localization and the recruitment to its target genes^40–42^. Here, we report a novel regulatory model that palmitic acid as one of the essential diet-derived metabolites directly enhances transcriptional activity of c-Myc via dual palmitoylation-dependent pathways, which emphasizes the importance of modulating effects of metabolites on transcriptional factors to orchestrate the interacting networks among metabolism, inflammatory response and tumorigenesis. This regulatory model might serve as a general mechanism by which metabolites manipulate other transcriptional factors.

Chronic colonic inflammation is significantly associated with the occurrence and malignant progression of colon cancer^43^. For example, long-term inflammatory bowel diseases (IBDs), such as Crohn’s disease and ulcerative colitis, are associated with a 1.4–2.2 times increased risk of colon cancer^44,45^. The sustained inflammation burden and repeated cycles of injury repair in the intestinal mucosa are linked to the generation of oxidative stress-induced damage to DNA. Meanwhile, complex and varied environmental factors further facilitate a sequence of genetic, epigenetic and transcriptional alterations, which promotes the carcinogenic process^43^. Elucidating the underlying mechanisms by which colonic inflammation facilitates c-Myc activation may assist to further deepen the mechanistic understanding of colon tumorigenesis. We firstly elucidated that palmitoyltransferase ZDHHC9 were upregulated by the inflammation factor IL-1β under colonic inflammation, and then lead to the hyperactivity of c-Myc transcriptional function. IL-1β has been described to regulate the expression of several proteins such as ST6GAL1 sialyltransferase through promoting the binding of NF-κB to the *ST6GAL1* promoter in pancreatic ductal adenocarcinoma^46^. It is further required to investigate the specific molecular mechanism of ZDHHC9 upregulation by inflammation factor IL-1β. The mechanism by which inflammation stimulates transcriptional activation of c-Myc through upregulation of palmitoyltransferases such as ZDHHC9 might occur in other inflammation-associated tumors, which is worth further investigating. Additionally, in the context of intestinal inflammation, although IL-1β is predominantly produced by recruited myeloid immune cells^47–49^, colon epithelial cells have also been reported to secrete IL-1β under specific conditions^50^. Thus, the specific cellular source and composition of IL-1β during PA-induced colitis warrant further investigation.

High-fat diet is well known to be closely associated with developing colonic inflammation and subsequent inflammation-associated tumorigenesis^51–55^. Studies suggest that high-fat diet might aggravate the colitis phenotype through promoting oxidative stress, mitochondrial dysfunction, disrupting bile acid homeostasis and disturbing immune cell balance^56^. Here, we demonstrated that palmitic acid, a major component of high-fat diets, significantly exacerbates colonic inflammation. Emerging evidence highlights the potential role of palmitic acid to engage in multiple cell death pathways, such as apoptosis and necroptosis^57,58^. Several distinct mechanisms have been implicated in palmitic acid-induced cell death, such as inducing endoplasmic reticulum stress and oxidative stress^59,60^, triggering mitotic catastrophe through increasing p53 stability^61^, and promoting RIPK1 palmitoylation and SUMOylation to enhance its kinase activity^62,63^. Additionally, studies also indicated that palmitoylation was involved in regulating pyroptosis, another form of cell death^64^. Therefore, we hypothesize that palmitic acid-induced colonic inflammation likely involves multiple mechanisms, which warrants further investigation.

Although a large amount of research evidence has fully demonstrated the strong potential of c-Myc as a therapeutic target for colon cancer, c-Myc protein has been regarded as an “undruggable” target. Here, we find that the activation of c-Myc is highly augmented by the metabolite palmitic acid. Furthermore, significantly increased levels of palmitic acid were observed in tumor tissues obtained from patients with colon cancer^8^. These results potentially suggest metabolic addiction to palmitic acid as a promising therapeutic target to effectively interfere c-Myc transcriptional hyperactivity. Our research illustrates the molecular process of metabolic addiction to palmitic acid in colon cancer which involves the coordination of ZDHHC9 and FATP2. Thus, based on the vulnerability of palmitic acid metabolism in colon cancer, targeting ZDHHC9 and FATP2 exerts promising therapeutic efficacy against colon cancer, which is supported by our in vivo studies. However, it remains to be investigated whether other ZDHHC family members, beyond ZDHHC9, can regulate the palmitoylation of c-Myc, particularly in cancer types other than colon cancer. Furthermore, as no specific inhibitors for ZDHHC9 are currently available, future efforts could focus on developing agents that disrupt its enzymatic activity or its interaction with c-Myc.

In summary, we have comprehensively demonstrated the molecular process of abnormal palmitic acid metabolism promoting c-Myc transcriptional activation underlying inflammation-associated colon tumorigenesis, proposing a novel model of metabolites in regulating transcription factors. Furthermore, this study brings up a promising therapeutic strategy targeting ZDHHC9 and FATP2 against colon cancer.

## Materials and methods

### Animals

Male C57BL/6 mice at 8 weeks old were purchased from Beijing Charles River Laboratory Animal Technology Co., Ltd. (Beijing, China) and female nude mice at 4–5 weeks old were purchased from Hangzhou Ziyuan Experimental Animal Technology Co., Ltd. (Hangzhou, China). All animals were housed under SPF conditions and animal care was provided in accordance with the institutional guidelines. All animal studies were approved by the Animal Research Committee at Zhejiang University (IACUC-25-S251).

### Establishment of mice colitis model

For the establishment of chronic colitis model, male C57BL/6 mice at 8 weeks old were administrated with 1.5% DSS (w/v, Yeasen Biotechnology Co. Ltd., Shanghai, China) diluted in drinking water for 7 days, following 7 days of untreated drinking water. This treatment cycle was repeated three times and followed by 7 days of 1.5% DSS solution. The entire experimental period is 50 days in total. The 7.5% PA diet mimics the PA contents in classical high-fat diets (5.7%–8.5%, by weight). In 15% PA diet, PA almost serves as the sole lipid source to replace all fats in a classical high-fat diet, while maintaining an equivalent fat-derived caloric fraction (45% Kal). Meanwhile, mice were randomly subjected to 4 groups fed with different diets: 7.5% PAD (*n* = 5, 7.5% PAD contains 7.5% (w/w) palmitic acid with 26.6% Kcal fat, customized by SYSE Biotechnology Co. Ltd, Changzhou, China), 15% PAD (*n* = 5, 15% PAD contains 15% (w/w) palmitic acid with 45% Kcal fat, customized by SYSE Biotechnology Co. Ltd.), HFD (*n* = 5, HFD contains 19.5% (w/w) lard with 45% Kcal fat, SYSE Biotechnology Co. Ltd) and ND as control (*n* = 5, ND contains 12.5% Kcal fat supplemented with soybean oil). During the experimental period, disease activity index (DAI) was used to quantitatively evaluate colitis symptoms.

### Subcutaneous xenograft model

Female nude mice at 4–5 weeks old were supplied with ND or 7.5% PAD for two weeks in advance (and supplied throughout the whole experimental period). Then 1 × 10^6^ HCT116 cells were injected subcutaneously into these nude mice. The volume of tumors was daily measured and calculated as (length × width^2^/2). After the tumors reached 50–100 mm^3^, mice were randomly divided into 7 groups (*n* = 7) and intraperitoneally administered with saline, 2BP (50 mg/kg), 2BP (100 mg/kg), Lipofermata 2 mg/kg, Lipofermata 4 mg/kg and 2BP (50 mg/kg) combined with Lipofermata (2 mg/kg) once a day. After 15-day treatment, the animals were sacrificed and the xenografts were weighed and photographed. Animals would be dropped from the study and euthanised before the predetermined time point if the size of a subcutaneous tumour exceeds 2000 mm^3^.

### Cell culture

Human SW620, HCT116 and HEK-293T cells along with Africa green monkey kidney fibroblast cell COS-7 were purchased from the Shanghai Institute of Biochemistry and Cell Biology (Shanghai, China). Cells were maintained in medium with 1% penicillin/streptomycin at 37 °C with 5% CO_2_. HCT116, COS-7 and HEK-293T cells were cultured in DMEM medium (Gibco, NY, USA) with 10% (v/v) fetal bovine serum (Gibco, NY, USA). SW620 cells were cultured in RPMI-1640 medium (Gibco, NY, USA) with 10% (v/v) fetal bovine serum.

### Cellular proliferation and colony formation assay

For cell proliferation assay, cells were seeded into 96-well plates (1000–2000 cells per well) and then subjected to SRB staining at the experimental endpoint. For colony formation assay, cells were cultured in 6-well plates (1000–2000 cells per well) for 14 days and subjected to SRB staining. Tris base aqueous solution was added to dissolve SRB dye and the absorbance at 515 nm was measured using a multifunctional microplate detector.

### Sphere formation assay

Cells were seeded into non-adherent 24-well plates and cultured in DMEM/F12 medium (Gibco, NY, USA) supplemented with 20 ng/mL EGF (Beyotime, Shanghai, China), 10 ng/mL FGF (Beyotime, Shanghai, China) and 0.4% (w/v) bovine serum albumin (Yeasen, Shanghai, China). The bright-field images of spheres were acquired by an Olympus microscope.

### Palmitoylation detection

The palmitoylation of c-Myc protein was detected by click chemistry and ABE assay. Details for palmitoylation detection are provided in Supplementary Information.

### Measurement of palmitic acid levels

The measurement of PA levels in tissues was performed at Wuhan Metware Biotechnology Corporation.

### Transcriptome sequencing

RNA sequencing was performed at Shanghai Biotechnology Corporation. Briefly, total RNA was isolated using RNeasy mini kit (Qiagen, Germany). The library construction and sequencing were performed at Shanghai Biotechnology Corporation. Agilent 4200 Bioanalyzer was employed to evaluate the concentration and size distribution of cDNA library before sequencing with an Illumina Novaseq 6000. The raw reads were filtered by Seqtk before mapping to genome using Hisat2 (version:2.0.4). The fragments of genes were counted using stringtie (v1.3.3b) followed by TMM (trimmed mean of M values) normalization. FastQC (v0.11.9) was used for quality check. Significant differentially expressed genes (DEGs) were analyzed using edgeR software. Raw data for RNA sequencing are deposited at the NCBI GEO (GSE299569).

## Supplementary information

Supplementary information, Figures and Tables

## Data Availability

The data that support the findings of this study are available from the corresponding author upon reasonable request.
