# Supplementary material for: Palmitic acid activates c-Myc via dual palmitoylation-dependent pathways to promote colon cancer
Source: Cell Discov. 2026 Feb 17;12:12. doi: 10.1038/s41421-026-00869-6 (PMC12909841; doi:10.1038/s41421-026-00869-6)
Supplement: Supplementary file 1 — Supplementary information, Figures and Tables [file 41421_2026_869_MOESM1_ESM.pdf]

Supplemental Information

Supplementary Fig.S1

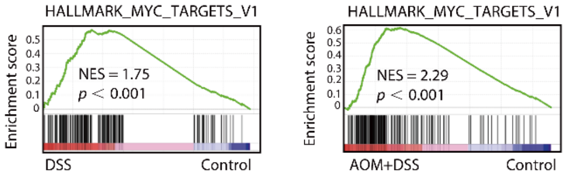

Supplementary Fig. S1: Gene set enrichment analysis (GSEA) in the DSS-induced colitis model (GSE208395) and murine model of inflammation-driven tumorigenesis treated with AOM and DSS (GSE249124).

Supplementary Fig.S2

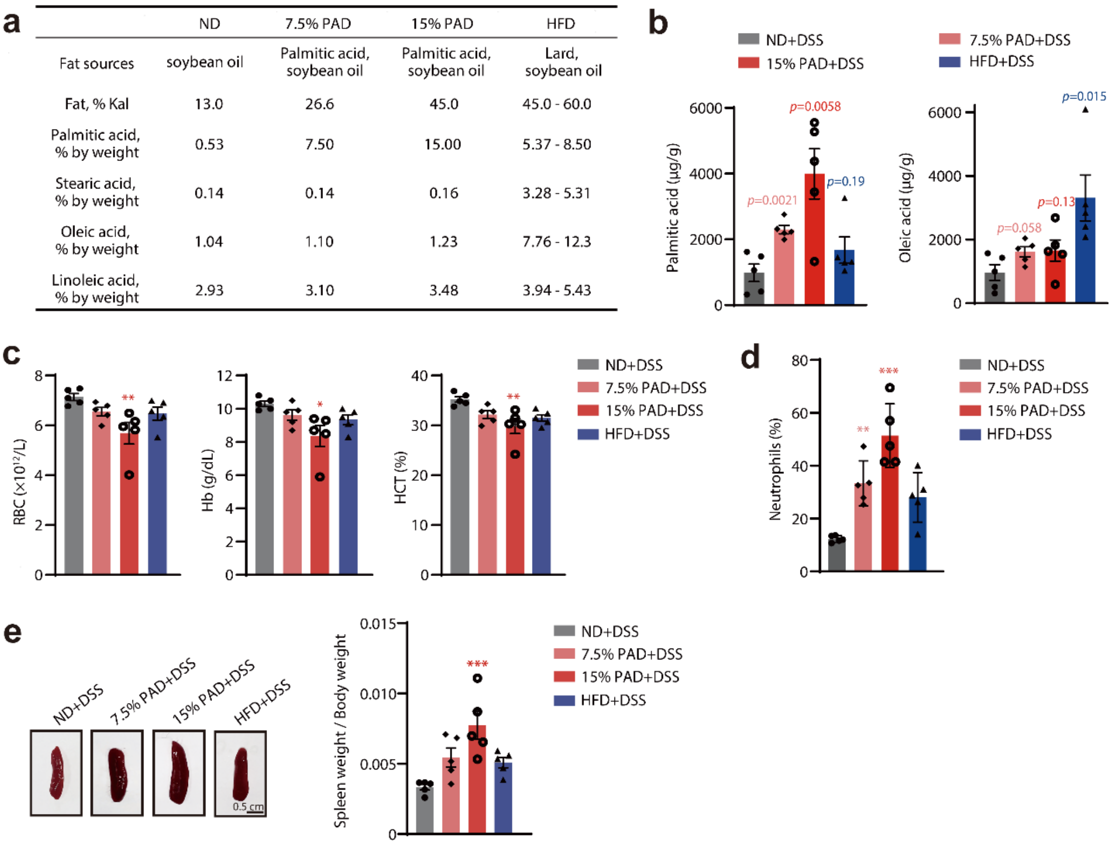

	ND	7.5% PAD	15% PAD	HFD
Fat sources	soybean oil	Palmitic acid, soybean oil	Palmitic acid, soybean oil	Lard, soybean oil
Fat, % Kal	13.0	26.6	45.0	45.0 - 60.0
Palmitic acid, % by weight	0.53	7.50	15.00	5.37 - 8.50
Stearic acid, % by weight	0.14	0.14	0.16	3.28 - 5.31
Oleic acid, % by weight	1.04	1.10	1.23	7.76 - 12.3
Linoleic acid, % by weight	2.93	3.10	3.48	3.94 - 5.43

Supplementary Fig. S2: The effect of palmitic acid diet in the colon inflammation of mice when exposed to DSS. a Diet features of ND, 7.5% PAD, 15% PAD and HFD. **b** The content of palmitic acid and oleic acid in colonic tissues derived from mice exposed to DSS fed with ND, 7.5% PAD, 15% PAD and HFD. **c** Red blood cell (RBC) count, hemoglobin (Hb) level and hematocrit (HCT) in peripheral blood samples of mice exposed to DSS fed with ND, 7.5% PAD, 15% PAD and HFD. **d** The proportion of neutrophils in peripheral blood samples. **e** Representative images of the spleen in each group. The spleen organ coefficients were calculated.

Supplementary Fig.S3

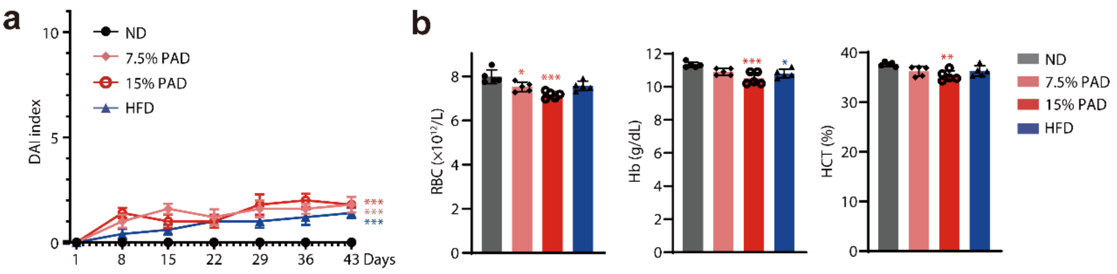

Supplementary Fig. S3: The effect of palmitic acid diet alone in the colon inflammation of mice. a DAI in mice fed with ND, 7.5% PAD, 15% PAD and HFD. **b** Red blood cell (RBC) count, hemoglobin (Hb) level and hematocrit (HCT) in peripheral blood samples of mice fed with ND, 7.5% PAD, 15% PAD and HFD.

Supplementary Fig.S4

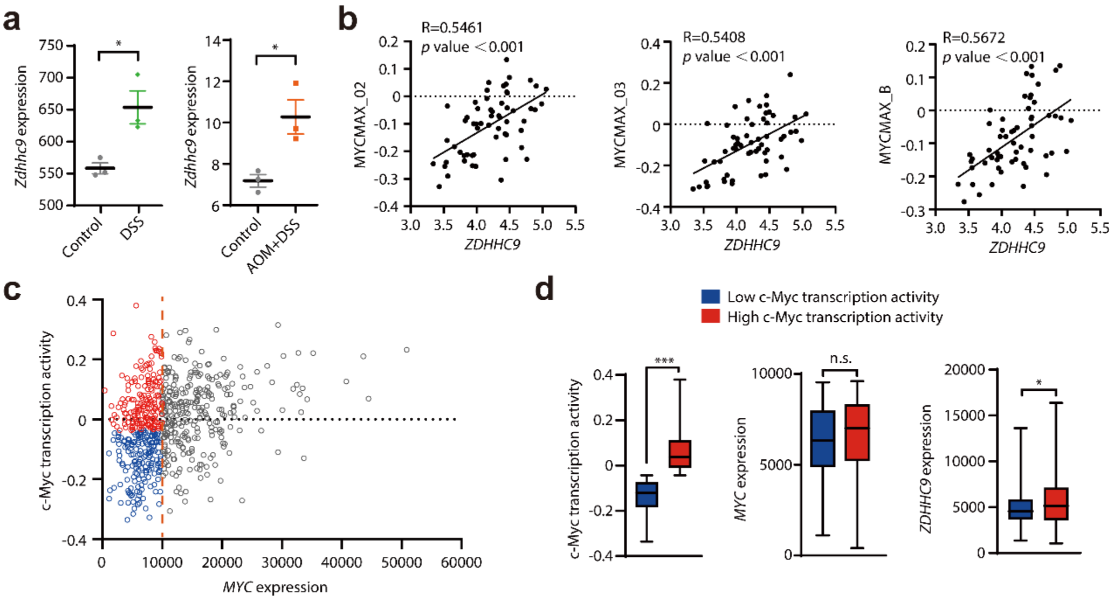

Supplementary Fig. S4: The correlation between ZDHHC9 and c-Myc in colon cancer. a Analysis of *Zdhhc9* expression in DSS-induced murine colitis model (GSE208395) and inducible murine model of inflammation-driven tumorigenesis treated with AOM and DSS (GSE249124). **b** Pearson correlation analysis of *ZDHHC9* expression with c-Myc transcriptional activity indicated by the GSVA score of c-Myc target gene sets in adenoma samples from GSE117606 dataset. **c** The correlation of MYC expression with c-Myc transcriptional activity, as indicated by GSVA score of MYCMAX_02 gene set, in colon cancer samples from TCGA database. Red dots indicated the colon cancer samples with relative low expression levels of *MYC* (*MYC* count < 10000) and relative higher c-Myc transcriptional activity (n=171). Blue dots indicated the colon cancer samples with relative low expression levels of *MYC* (*MYC* count < 10000) and relative lower c-Myc transcriptional activity (n=171). **d** Left, the c-Myc transcriptional activity in these two groups. Middle, the expression levels of *MYC* in these two groups. Right, the expression levels of *ZDHHC9* in these two groups.

Supplementary Fig.S5

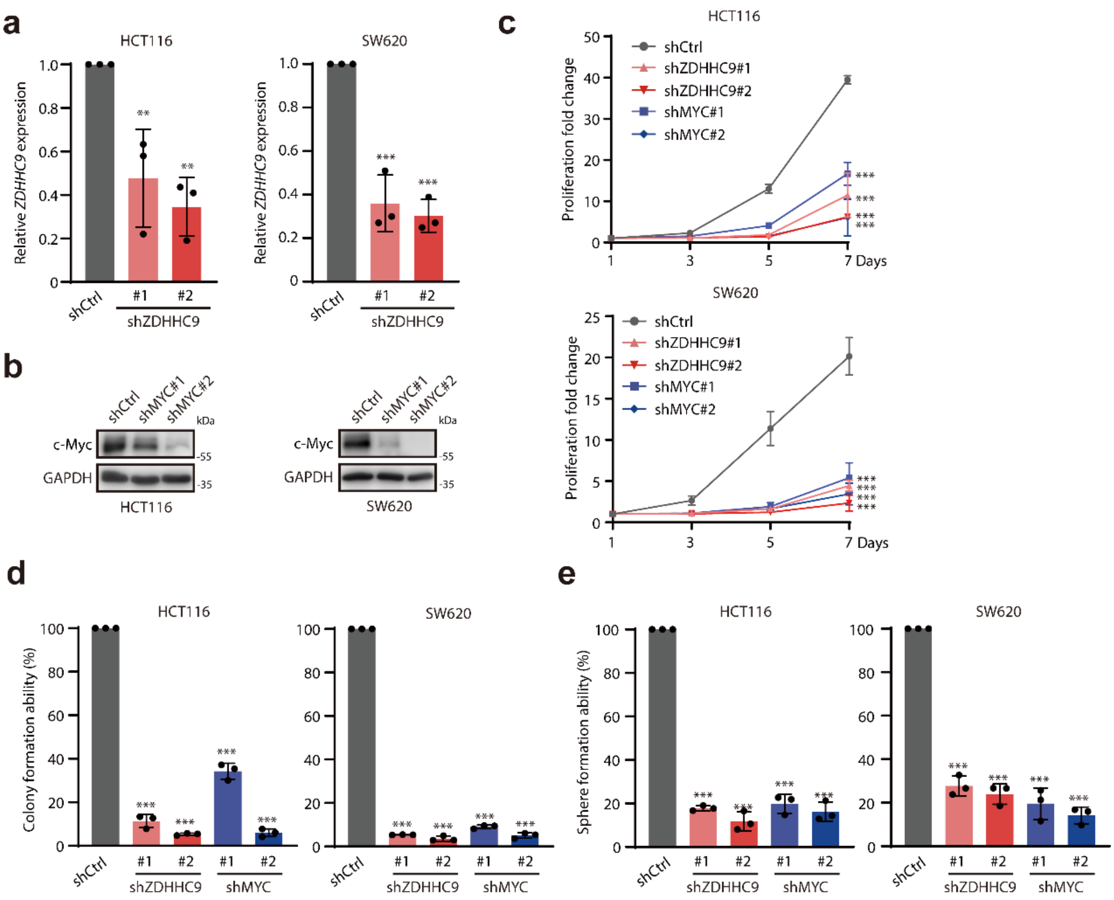

Supplementary Fig. S5: The effect of shZDHHC9 on the proliferation and clone formation of colorectal cancer cells. a The silencing efficiency of different shRNAs targeting ZDHHC9 in HCT116 and SW620 cells measured by RT-PCR. **b** The silencing efficiency of different shRNAs targeting MYC in HCT116 and SW620 cells measured by western-blotting. **c** The cell proliferation assay of colon cancer cells HCT116 and SW620 infected with lentivirus-shZDHHC9 or shMYC for 7 days, as evaluated by SRB staining. **d** The colony formation assay showing the suppressed colony formation capacity caused by ZDHHC9 or MYC knockdown in colon cancer cells HCT116 and SW620. **e** The sphere formation assay showing the impaired sphere formation ability caused by ZDHHC9 or MYC knockdown in colon cancer cells HCT116 and SW620.

Supplementary Fig.S6

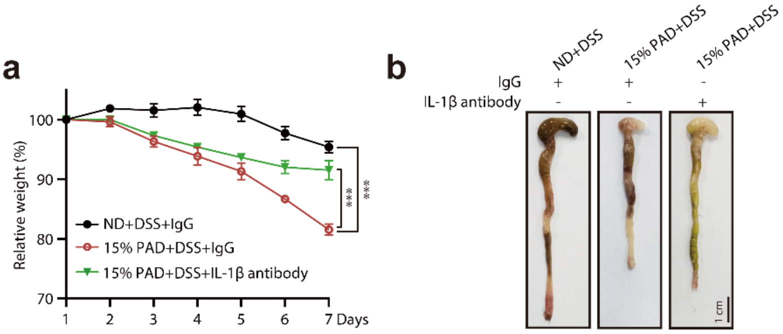

Supplementary Fig. S6: The effect of IL-1β neutralizing antibody on palmitic acid diet-induced colitis in mice. a The relative weight of mice exposed to 2% DSS-induced colitis respectively fed with ND, 15% PAD and subjected to administration of IgG or IL-1β antibody (10 mg/kg, *i.p.*). **b** Representative images of the colon tissue in each group.

Supplementary Fig.S7

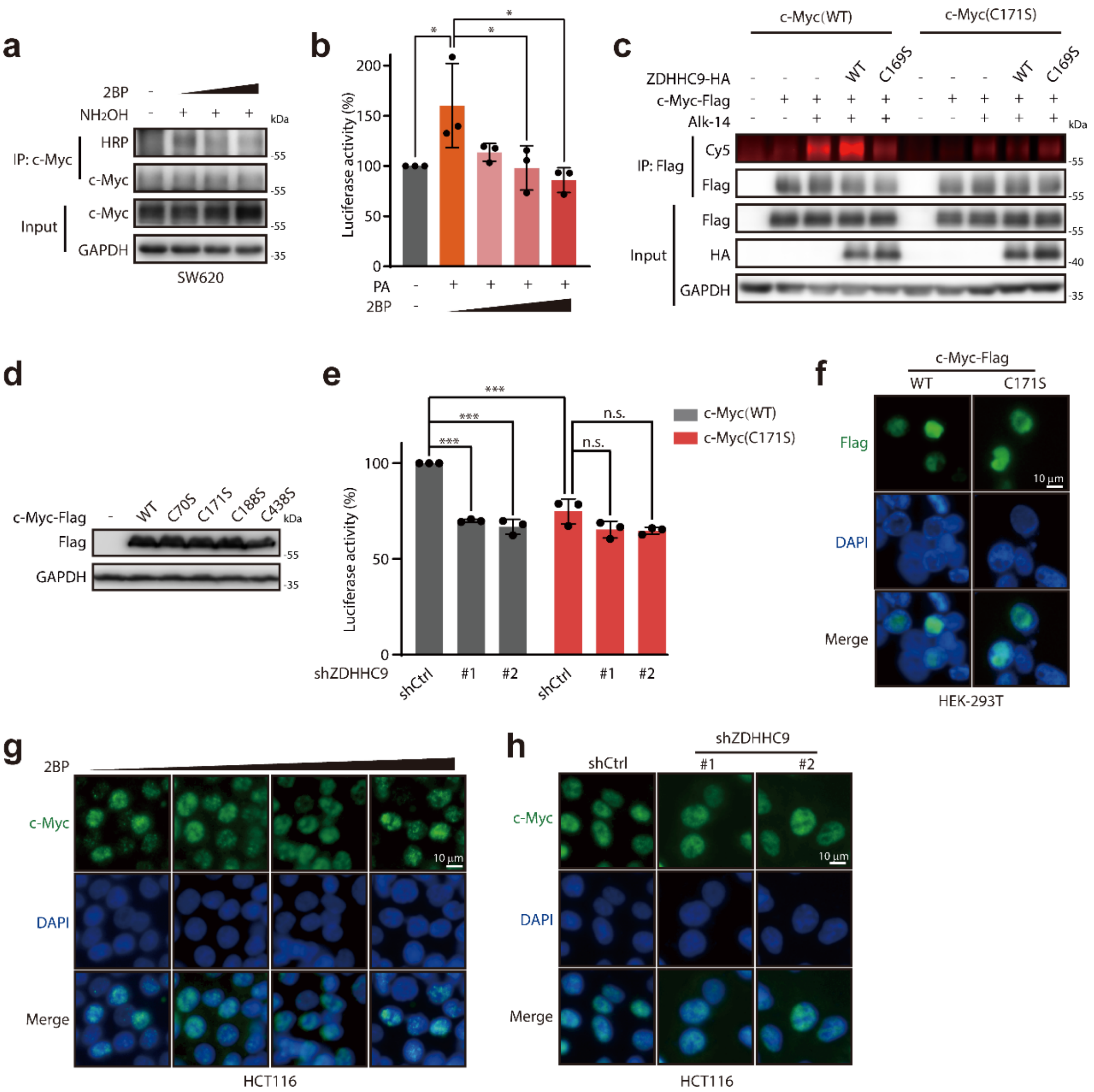

Supplementary Fig. S7: The influence of palmitoylation of c-Myc on its transcriptional

activity and localization. a ABE assay showed the inhibition of pan-palmitoyltransferase inhibitor 2BP (20 μM or 40 μM) on the palmitoylation level of c-Myc protein in SW620 cells. **b** The inhibitory effect of 2BP (20 μM, 40 μM or 80 μM) on c-Myc transcriptional activity promoted by palmitic acid (25 μM). **c** In-gel fluorescence exhibited the effect of ZDHHC9-wild type and ZDHHC9-C169S on the palmitoylation level of c-Myc wild type or C171S mutant. **d** The protein levels of c-Myc wild type as well as C70S, C171S, C188S, and C438S mutant in HEK-293T cells. **e** The effects of ZDHHC9 knockdown on the transcriptional activity of c-Myc wild type or C171S mutant. **f** IF staining of cells for c-Myc wild type and C171S mutant in HEK-293T cells. **g** IF staining of cells for c-Myc in HCT116 cells treated with 2BP (20 μM, 40 μM or 80 μM). **h** IF staining of cells for c-Myc in HCT116 cells infected with shCtrl, shZDHHC9#1 or shZDHHC9#2 lentivirus.

Supplementary Fig.S8

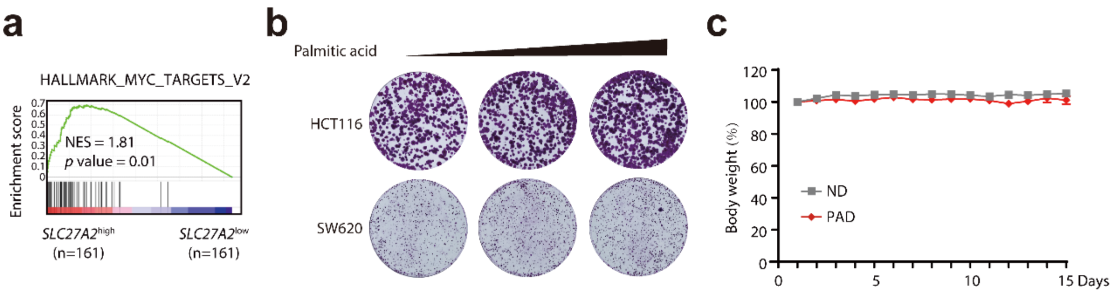

Supplementary Fig. S8: The promoting effect of palmitic acid on colorectal cancer. a GSEA plot depicting the enrichment of c-Myc target genes in colon cancer samples with relatively higher *SLC27A2* expression. **b** Representative images exhibiting the colony formation of colon cancer cells HCT116 and SW620 cultured in mediums containing fetal bovine serum with the treatment of palmitic acid (3.13 μM or 6.25 μM). **c** The relative body weight of nude mice supplied by ND or 7.5% PAD.

Supplementary Fig.S9

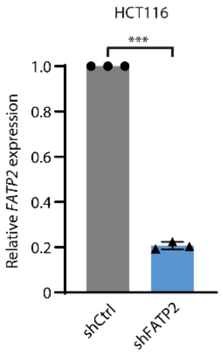

Supplementary Fig. S9: The silencing efficiency of shRNA targeting FATP2 in HCT116 measured by RT-PCR.

Supplementary Fig.S10

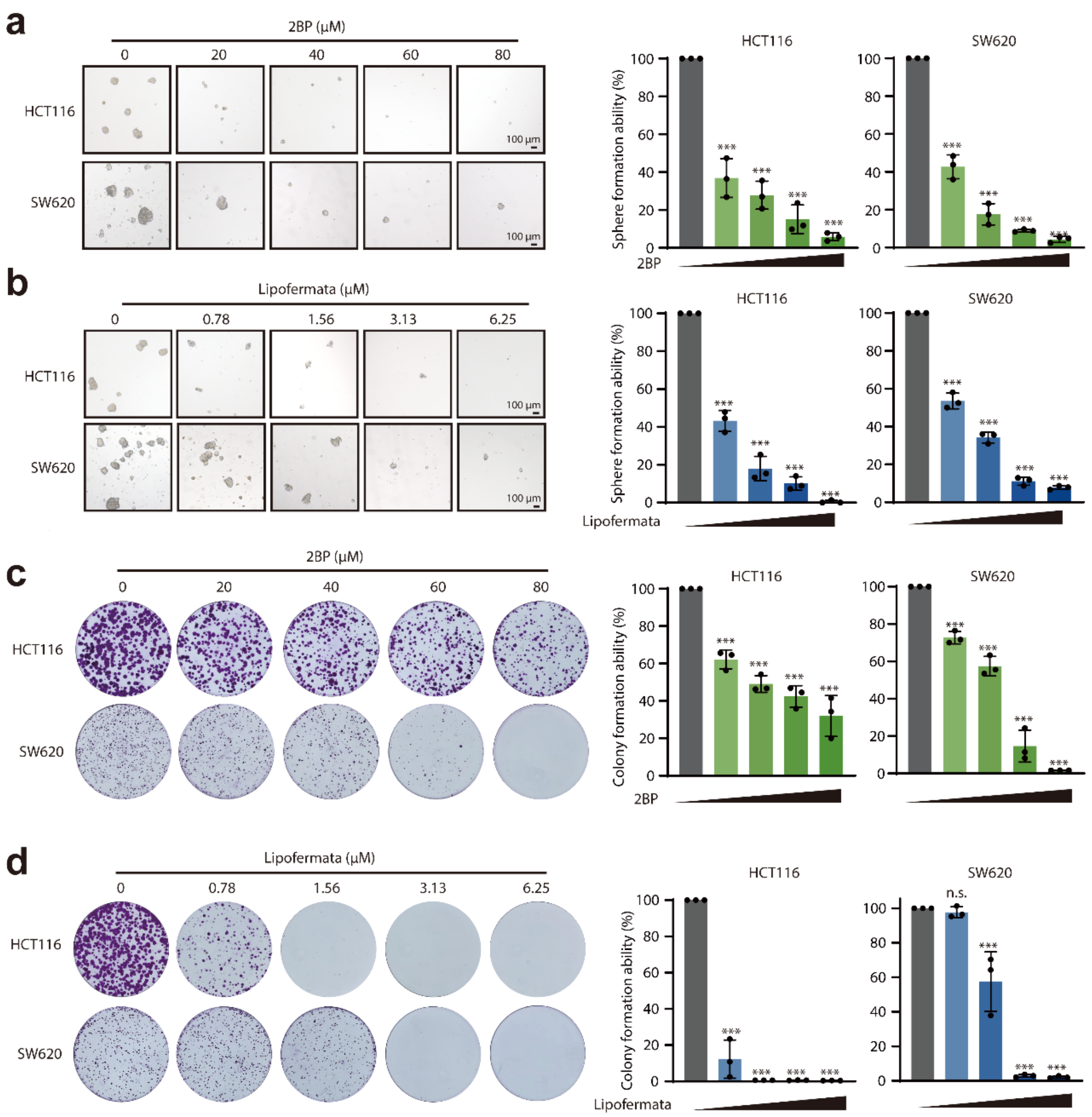

Supplementary Fig. S10: The effect of 2BP and Lipofermata on sphere and clone formation in colorectal cancer cells. a Sphere formation assay of colon cancer cell HCT116 and SW620 treated with 2BP. **b** Sphere formation assay of colon cancer cell HCT116 and SW620 treated with Lipofermata. **c** Colony formation assay of colon cancer cell HCT116 and SW620 treated with 2BP. **d** Colony formation assay of colon cancer cell HCT116 and SW620 treated with Lipofermata.

Supplementary Fig.S11

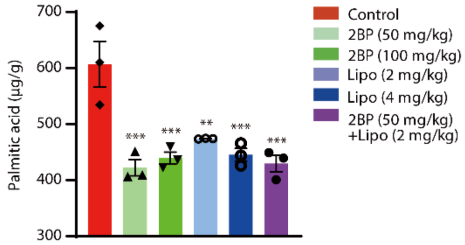

Supplementary Fig. S11: The content of palmitic acid and in HCT116 xenografts derived from nude mice fed with PAD treated with 2BP and Lipofermata.

Method details

Reagents, antibodies and plasmids

Lipofermata was purchased from MedChemExpress (Princeton, USA). Palmitic acid and 2BP were purchased from Sigma-Aldrich (St. Louis, MO). Z-IED-FMK was purchased from Selleck. 2-Bromotetradecanoic acid and 2-Bromooctadecanoic acid were purchased from Shanghai Bide Pharmaceutical Technology Co., Ltd (Shanghai, China). 12-Bromododecanoic acid was purchased from Shanghai Yuanye Biotechnology Co., Ltd (Shanghai, China). All agents were dissolved in DMSO.

Antibody against c-Myc (Catalog No. 5605S, Clone No. D84C12) and DYKDDDDK (Catalog No. 14793S, Clone No. D6W5B) were purchased from Cell Signaling Technology. Antibody against HA (Catalog No. Db2603) and GAPDH (Catalog No. Db106) were purchased from Hangzhou Daige Biotechnology Co., Ltd (Hangzhou, China). Antibody against MAX (Catalog No. 10426-1-AP) were purchased from Proteintech Group, Inc (Wuhan, China).

Murine *ZDHHC9* plasmid was kindly provided by Dr. Hening Lin. Murine *ZDHHC9* C169S mutant plasmid was obtained through point mutation. The human *MYC* was amplified from the HEK-293T cDNA library and subcloned into a pCDNA3.0 plasmid. *MYC* C70S, C171S, C188S and C438S mutant plasmids were respectively obtained through point mutation. The shRNA oligonucleotides targeting *ZDHHC9*, *MYC* and *FATP2* were annealed and cloned into pLKO.1 vector, shRNA sequences were shown in **Supplementary Table S1**.

Supplementary Table S1: The list of shRNA sequences

shRNA	sequences
shZDHHC9#1	GAGGAACTACCGCTACTTCTA
shZDHHC9#2	CTGTTACACATGCAAGATCTT
shMYC#1	CAGTTGAAACACAAACTTGAA
shMYC#2	CCTGAGACAGATCAGCAACAA
shFATP2#1	CCTATGACTGAGGACATCTAT

Establishment of Mice Colitis Model and experimental procedure

(1) DAI evaluation

During the experimental period, disease activity index (DAI) was used to quantitatively evaluate colitis symptoms. DAI score = Body weight loss score + Fecal occult blood score + Fecal viscosity score, and specific evaluation criteria were displayed in **Supplementary Table S2-4**. Fecal blood was examined by fecal occult blood test kit (Nanjing Jiancheng Bioengineering Institute, Nanjing, China).

Supplementary Table S2: Body weight loss percentage scoring criteria

Score	Body weight loss percentage
0	0%
1	1-5%
2	5-10%
3	10-20%
4	＞20%

Supplementary Table S3: Fecal viscosity scoring criteria

Score	Fecal viscosity
0	Normal
1	Soft
2	Mucous
3	Loose

Supplementary Table S4: Fecal occult blood scoring criteria

Score	Fecal occult blood
0	No color change after 3 minutes of reaction
1	Reacts to light blue-green at 30 seconds and turns to blue-green at 60 seconds
2	Reacts to light blue-green for 10 seconds, turns blue-green for 30 seconds
3	Reacts to blue-green within 10 seconds
4	Immediately reacts to blue-green
5	Visible bloody stool

(2) Histopathology analysis

Colon tissue was collected and quickly fixed with 4% paraformaldehyde and embedded in paraffin. Paraffin sections were cut and subsequently stained with hematoxylin and eosin. Histopathological scoring was performed as previously described[1].

(3) Blood test

Peripheral blood samples were collected to perform complete blood analysis for examining red blood counts ($\times 10^{12}$/L), hemoglobin (g/dL), hematocrit (%) and proportion of neutrophils (%).

Immunoprecipitation

For detecting the protein-protein interaction of c-Myc with ZDHHC9, co-Immunoprecipitation was performed as described previously[2].

Palmitoylation detection by click chemistry

Cells were incubated with 100 nM Alk-14 for 8 hours before collection. Then cells were collected and lysed in lysis buffer containing 4% SDS to obtain protein samples. The target proteins were pulled down through immunoprecipitation and subjected to click chemistry reaction. Beads were suspended by 20 μL RIPA buffer with 0.5 mM TBTA, 2.5 μM $CuSO_4$, 1 mM TCEP and 0.1 mM Cy5.5 dye for click chemistry reaction. After 30 minutes of incubation, the reaction was terminated by adding 10 μL loading buffer and subjected to SDS-PAGE. The in-gel Cy5.5 signaling was detected by Amersham Typhoon.

Palmitoylation detection by ABE assay

Cells were collected and lysed in lysis buffer containing 4% SDS then target proteins were pulled down through immunoprecipitation. The immunoprecipitated proteins on beads were treated with 1 mM N-Ethylmaleimide (pH 7.4), which irreversibly binds to non-palmitoylated cysteine residues preventing from reacting with subsequent reagents. Subsequently the proteins on beads were subjected to the treatment with 1 M hydroxylamine (NH_2OH, pH 7.2), in order to selectively hydrolyze thioester bonds, releasing palmitic acid and exposing the thiol group of the previously palmitoylated cysteine. Then proteins were reacted with 5 μM Biotin-BMCC (pH 6.8) to specifically label the palmitoylation sites after NH_2OH treatment. After elution, the proteins were subjected to SDS-PAGE and western-blotting. The palmitoylated proteins were detected by HRP.

Quantitative Real-time PCR

Real-time PCR was performed to measure the mRNA levels of *ZDHHC9,* as described previously[3]. *GAPDH* transcripts serve as internal control. The primers used for real-time PCR are shown in **Supplementary Table S5**.

Supplementary Table S5: Quantitative Real-time PCR Primers

Primer	Sequence
ZDHHC9-F	AGAATCGCGTCCAGAATCCC
ZDHHC9-R	TTCCACTTTCCTCCAGTGGC
GAPDH-F	CCAGCAAGAGCACAAGAGGA
GAPDH-R	ACATGGCAACTGTGAGGAGG
SLC27A2-F	ATGTGGCCACCACTGAAGTT
SLC27A2-R	GGGTTAAAGCCCTCCTCCAC
ASPSCR1-F	TTGTTCCTTTCTCGGGTGGG
ASPSCR1-R	ACTTCTTTGGCTTGGAGGGG
CTSK-F	CCCGCAGTAATGACACCCTT
CTSK-R	AAAGCCCAACAGGAACCACA
CAMKK1-F	CCCCGAGGCCATTTCTGATT
CAMKK1-R	AAACACCACGGGCTCATTCT
Gapdh-F	GCGAGACCCCACTAACATCA
Gapdh-R	CATGAGCCCTTCCACAATGC
Zdhhc9-F	TGGACAGGGAAGAATCGTGTG
Zdhhc9-R	TCAGGAATGCTGGTGTCCTC
Myc-F	CAGCTCGCCCAAATCCTGTA
Myc-R	GTGTCTCCTCATGCAGCACT
Bmi1-F	TTACGATGCCCAGCAGCAAT
Bmi1-R	GCTCTCCAGCATTCGTCAGT
Ilf3-F	TGTGTGAGAAGTCCATCGGC
Ilf3-R	CCACCGGGTTCTCGTTCTTT

The detection of cytokine protein levels

The protein levels of cytokines in Figure 3A were measured by the glass slide-based Mouse Inflammation cytokine array (GSM-INF-1, Raybiotech, Georgia, USA).

Statistical analysis

Cell-based experiments were performed with three biological replicates and data are presented as mean ± SD. For animal study, data are presented as mean ± SEM. Statistical significance of differences between groups was determined by unpaired two-tailed Student's t-

test analysis, Tukey tests as part of one way and two-way ANOVA, or Log-rank test through GraphPad Prism. Statistical test used for a particular experiment is mentioned in figure legends.

Reference

1. Meira, L.B. *et al.* DNA damage induced by chronic inflammation contributes to colon carcinogenesis in mice. *Journal of Clinical Investigation* **118**, 2516-2525 (2008).
2. Shao, X.J. *et al.* Blockade of deubiquitinase YOD1 degrades oncogenic PML/RARα and eradicates acute promyelocytic leukemia cells. *Acta Pharmaceutica Sinica B* **12**, 1856-1870 (2022).
3. Du, W.X. *et al.* A novel gene fusion RUNX1/ZNF423 promotes leukemic relapse of NUP98-rearranged AML. *Leukemia* **37**, 2286-2291 (2023).
